# IL1- and TGFβ-Nox4 signaling, oxidative stress and DNA damage response are shared features of replicative, oncogene-induced, and drug-induced paracrine ‘Bystander senescence’

**DOI:** 10.18632/aging.100520

**Published:** 2012-12-30

**Authors:** Sona Hubackova, Katerina Krejcikova, Jiri Bartek, Zdenek Hodny

**Affiliations:** ^1^ Department of Genome Integrity, Institute of Molecular Genetics, v.v.i., Academy of Sciences of the Czech Republic, Prague, Czech Republic; ^2^ Danish Cancer Society Research Center, Copenhagen, Denmark

**Keywords:** senescence-associated secretome, DNA damage response, cytokines, JAK/STAT3, TGFβ, NFκB, IL6, IL1β, Nox4, autocrine and paracrine signaling, tumor microenvironment

## Abstract

Many cancers arise at sites of infection and inflammation. Cellular senescence, a permanent state of cell cycle arrest that provides a barrier against tumorigenesis, is accompanied by elevated proinflammatory cytokines such as IL1, IL6, IL8 and TNFα. Here we demonstrate that media conditioned by cells undergoing any of the three main forms of senescence, i.e. replicative, oncogene- and drug-induced, contain high levels of IL1, IL6, and TGFb capable of inducing reactive oxygen species (ROS)-mediated DNA damage response (DDR). Persistent cytokine signaling and activated DDR evoke senescence in normal bystander cells, accompanied by activation of the JAK/STAT, TGFβ/SMAD and IL1/NFκB signaling pathways. Whereas inhibition of IL6/STAT signaling had no effect on DDR induction in bystander cells, inhibition of either TGFβ/SMAD or IL1/NFκB pathway resulted in decreased ROS production and reduced DDR in bystander cells. Simultaneous inhibition of both TGFβ/SMAD and IL1/NFκB pathways completely suppressed DDR indicating that IL1 and TGFβ cooperate to induce and/or maintain bystander senescence. Furthermore, the observed IL1- and TGFβ-induced expression of NAPDH oxidase Nox4 indicates a mechanistic link between the senescence-associated secretory phenotype (SASP) and DNA damage signaling as a feature shared by development of all major forms of paracrine bystander senescence.

## INTRODUCTION

Cancer incidence in humans sharply increases with advancing age. The reason for this is thought to be multifactorial, including aging related accumulation of mutations in cellular tumor-suppressive and tumor-promoting (oncogenic) pathways and age-related disturbance of immune surveillance. Importantly, these phenomena may be causally linked to systemic escalation of chronic inflammatory reactions known to increase with age [1, 2], as inflammation *per se* may lead to genotoxic effects [3] and immune system disturbance [4], thereby triggering a vicious circle of amplification of cancer permissive conditions in the organism.

Cellular senescence fueled by DNA damage checkpoints is regarded as a tumorigenesis barrier that prevents division of cells with damaged genomes [5, 6]. On the other hand, persistence of senescent cells in tissues is thought to be deleterious due to substances produced by senescent cells themselves [7, 8]. Half a century after Leonard Hayflick's proposal of the limited proliferative potential concept [9], accumulating evidence supports the contribution of senescent cells to organismal aging [10] and tumor-promoting properties of senescent cells under conditions when their clearance by immune system is compromised [11]. Given the fact that senescence-associated cell cycle arrest is not fully irreversible, at least in case of cancer senescent cells manipulated *in vitro* [12] [13-17], persistence of senescent cells in tissues might also represent a potential threat of senescence bypass and transition of senescent cell escapers with irreparable DNA damage into malignant cells.

Changes in gene expression characteristic for various forms of senescence are accompanied by a robust increase of mRNA and secretion of numerous cytokines, chemokines, growth factors and proteases [18-25]. This phenomenon was termed senescence-associated secretory phenotype (SASP; [26]) or senescence messaging secretome (SMS; [27]). Regulation at transcriptional and translational [28] levels contribute to SASP induction. As the SASP results primarily from genomic damage response, one of its beneficial functions might be to communicate with cells of the immune system through secretion of pro-inflammatory cytokines, especially TNFα, IL6, IL8 and IL1β, to signal the presence of damaged cells bearing a potential risk of tumor development [29]. In addition, SASP has also been implicated in tissue regeneration after damage. Matrix metalloproteinases secreted by senescent cells in damaged tissues protect against accumulation of collagen and fibronectin, thereby preventing fibrosis [30, 31]. On the other hand, accumulation of senescent cells in old people or patients undergoing immunosuppresive chemotherapy may impair organ functions in an age-dependent manner [32] and lead to tissue damage reflecting increased signaling of pro-inflammatory cytokines by spread of oxidative stress due to mito-chondrial dysfunction in neighboring cells [33]. In fact, not only the local microenvironment pathology, but also a variety of chronic degenerative diseases as well as cancer can be induced by circulating pro-inflammatory cytokines like IL6 [34]. More than fifty cytokines involved in intercellular signaling are secreted at higher levels by senescent cells [35]. It was found that senescence-associated cytokines can also amplify the senescence phenotype in an autocrine manner [20, 21] [36].

The produced cytokines may also mediate the impact of ionizing radiation on senescence, as in vivo mouse experiments showed the presence of DNA damage in tissues distant from the irradiated field [37] resembling a radiation-linked phenomenon termed “bystander effect” [38]. Subsequent experiments with irradiated cells implicated ROS activation in bystander cells as a generator of DNA double strand breaks (DSB), which in turn activate a cascade of proteins involved in the DDR and can result in cell cycle arrest [39]. It was shown that DNA damage in *in vitro*-irradiated cells was also contributed by long-term exposure to stress-induced cytokines (primarily TGFβ), which can activate DDR and may induce growth arrest through ROS-dependent induction of DSB formation [40].

Several cytokines trigger enhanced ROS production and DNA damage-induced senescence upon long-term exposure of cultured cells, including interferons type I [41, 42] and type II [43], TNFα [44], IL6 [45], and TGFβ [46]. Given that senescent cells produce these cytokine species frequently in a simultaneous fashion, it is not unexpected that such DNA damage-promoting cytokine environment can induce senescent cells in their neighborhood by paracrine effects (‘bystander senescence’; [47]) as has been documented in several experimental settings [48, 49]. However, the mechanisms underlying bystander senescence are currently unclear.

In this study we focused on the following conceptually important questions: i) Is the capacity to induce SASP-associated bystander senescence a feature shared by cells undergoing various forms of ‘primary/parental’ senescence?; ii) Which cytokine species and/or signaling pathways are causally involved in bystander senescence? and iii) What is their link with potential DNA damage in such settings? We found that culture media conditioned by cells undergoing replicative, oncogene- and drug-induced primary senescence are all capable of inducing elevated ROS production and DNA damage in normal bystander cells, and trigger their transition into cellular senescence. Furthermore, experimental inhibition of IL1β/NFκB and TGFb/ SMAD signaling led to: a) decreased expression of NADPH oxidase Nox4; b) decreased ROS production and c) suppression of DDR in bystander cells, indicating that IL1β and TGFβ are critical components of SASP causally involved in bystander senescence.

## RESULTS

### DNA damage response (DDR) is activated in the vicinity of senescent cells by secreted factors

Given the potential tumor-promoting properties of senescent cells [7, 50], we asked whether senescent cells can induce DNA damage in neighboring proliferating cells. Non-senescent osteosarcoma U2OS cells stably transfected with green fluorescent protein (GFP) were mixed with drug-induced senescent U2OS cells (prepared by exposure to 10 μM BrdU and 10 μM DMA as previously described [24]) at a ratio 10:1, cultured together for 24 hours and then assessed for the presence of GFP and serine 139 phosphorylated histone H2AX (γH2AX) foci as a marker of formation of DNA DSBs [51, 52]. Notably, there was a significant increase in the number of γH2AX foci not only in cells in close contact with senescent cells but also in distant cells (Suppl. Fig. 1A). This result is consistent with reported paracrine DNA damage evoked in the presence of radiation-induced senescent cells [53].

**Figure 1:**
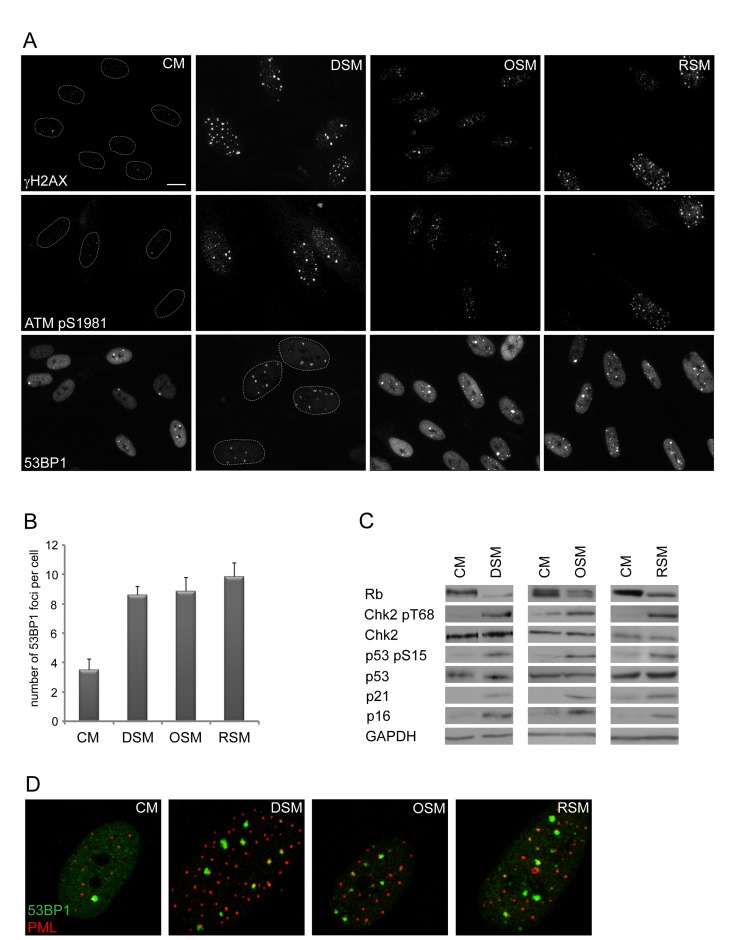
Conditioned medium from various types of senescent cells is able to induce DNA damage and DNA damage response in bystander cells (**A**) Immunofluorescence detection of 53BP1, γH2AX and serine 1981 phosphorylated ATM (ATMpS1981) in BJ cells treated with senescent medium from drug-induced (DSM), oncogene-induced (OSM) or replicative (RSM) senescent BJ cells for 20 days. BJ cells treated with medium from non-senescent BJ cells (CM) were used as a control; bar 15μm. (**B**) Quantification of numbers of 53BP1 foci in BJ cells treated with individual senescence-conditioned media. (**C**) Immunoblot detection of Rb, p21, p16, total p53 and Chk2, serine 15 phosphorylated p53 and threonine 68 phosphorylated Chk2 in BJ cells treated with different types of senescence-conditioned media. GAPDH was used as a loading control. (**D**) Immunofluorescence detection of PML and 53BP1 foci colocalization using confocal microscope in BJ cells with different types of senescent medium.

To analyze this phenomenon in more detail, we first asked whether cells undergoing senescence induced by any of the three major triggers: replication, activated oncogenes or genotoxic drugs possess analogous potential to induce DNA damage in neighboring cells. We exposed human normal BJ fibroblasts grown at relatively low passage (30 population doublings) to culture media partly enriched (1:1) by conditioned media of BJ cells brought to senescence either by genotoxic stress induced by etoposide (drug-induced senescence, DIS), activated H-Ras^V12E^ (oncogene-induced senescence; OIS; [54]) or exhaustion of replicative potential (replicative senescence; RS; population doubling 80; see Supplementary Figure 1B-E for characterization of “parental” senescent cells). Intriguingly, the exposure of ‘young’ BJ cells to any of the three types of senescence-conditioned media resulted in increased numbers of nuclear γH2AX foci. The elevation of γH2AX foci and total level of γH2AX was apparent from day 2 after transfer of cells to ‘senescent’ media and persisted at least to day 20 of continuous exposure as exemplified in Fig. 1A for DIS-BJ, RS-BJ and OIS-BJ conditioned media. Serine 1981 phosphorylated ATM, an active form of a kinase involved in serine 139 phosphorylation of H2AX [55], was also elevated in exposed BJ cells and accumulated in DNA damage nuclear foci, as well as 53BP1 (see Figs. 1A, B for foci quantification), another factor participating in DNA DSB sensing and repair [56]. Furthermore, increased levels of activated forms of two ATM substrates involved in activation of cell cycle checkpoints, checkpoint kinase Chk2 [57] and tumor suppressor p53 [58, 59], were detected in cells exposed to all three types of senescence-conditioned media followed from day 10 and continuing to day 20 (Fig. 1C) using antibodies against phospho-threonine 68 of Chk2 and phospho-serine 15 of p53, respectively. Note that the 53BP1/γH2AX nuclear foci co-associated with PML nuclear bodies (Fig. 1D), a feature characteristic for persistent DNA damage lesions [60-63], termed DNA-SCARS [64]. Besides normal human fibroblasts, we observed similar effects of DIS-conditioned medium inducing ‘paracrine’ DNA damage in U2OS cells (Suppl. Figs. 2A-C show that to induce drug-induced senescence in U2OS cells, a combination of 10 μM BrdU and 10 μM DMA were used, as previously described [24]). Clastogenic effect of the DIS secretome was further supported by appearance of enhanced micronucleation in U2OS cells exposed to senescent-conditioned medium (Suppl. Fig. 2D). Notably, no micronuclei were observed in any of the three types of bystander BJ cells.

Altogether, these data show that each of the three forms of SASP is capable of activating persistent DDR, both in human normal and cancer cells.

### DDR in ‘bystander’ cells is associated with development of cellular senescence

**Figure 2:**
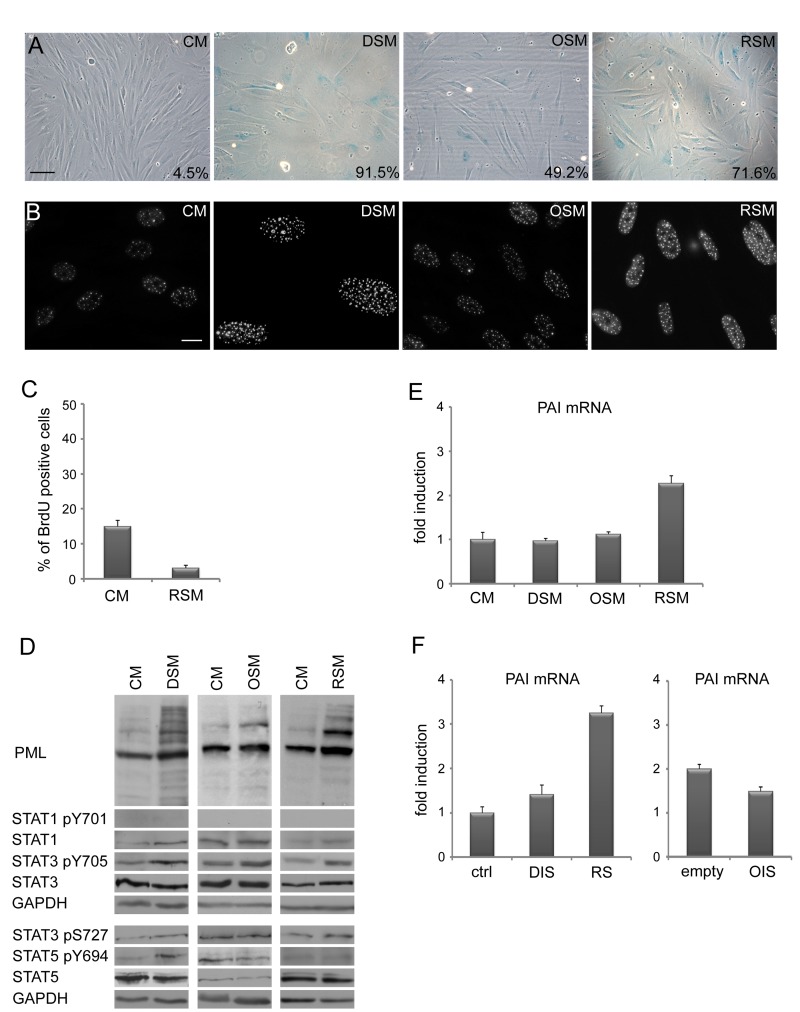
Medium from various types of senescent cells induces senescence in bystander cells (**A**) Senescence-associated β-galactosidase detection and (**B**) immunofluorescence detection of PML nuclear bodies (PML NBs) in BJ cells treated with senescent medium from drug-induced (DSM), oncogene-induced (OSM) or replicative (RSM) senescent BJ cells for 20 days. BJ cells treated with medium from non-senescent BJ cells (CM) were used as a control. Bar 100 μm for (**A**), 15 μm for (**B**). (**C**) Statistical analysis of BrdU incorporation in BJ cells treated for 4 days with conditioned medium from replicative senescent cells (RSM). BJ cells treated 4 days with medium from non-senescent cells (CM) were used as a control. (**D**) Immunoblot detection of PML, total STAT1, STAT3 and STAT5, STAT1 phosphorylated on tyrosine 701 (STAT pY701), STAT3 phosphorylated on tyrosine 705 and serine 727 (STAT3 p705 and pS727) and STAT5 phosphorylated on tyrosine 694 (STAT5 pY694). (**E**) Plasminogen activator inhibitor (PAI) mRNA levels quantified by real time qRT-PCR in BJ cells treated with senescent medium from drug-induced (DSM), oncogene-induced (OSM) or replicative (RSM) senescent BJ cells for 20 days. BJ cells treated with medium from non-senescent BJ cells (CM) were used as a control. The mRNA values represent average of two independent experiments and are shown as a fold induction relative to control BJ cells (CM); error bars represent standard error. β-actin was used as a reference gene. (**F**) PAI mRNA levels quantified by real time qRT-PCR in drug-induced (DIS), oncogene-induced (OIS) and replicative senescent (RS) BJ cells. The mRNA values represent average of two independent experiments and are shown as a fold induction relative to control BJ cells (ctrl) or BJ cells transfected with empty vector (empty); error bars represent standard error. β-actin was used as a reference gene.

As prolonged activation of DDR and cell cycle check-points result in permanent cells cycle arrest (cellular senescence; for a review, see [65, 66]), we next assessed the presence of senescent cells in cultures exposed to conditioned ‘senescent’ or control media using established markers of cellular senescence. Estimated at day 20 after exposure, all three types of senescence- conditioned media led to increased activity of senescence-associated-β-galactosidase (SA-β-Gal; [67]; Fig. 2A), elevated numbers and increased size of PML nucler bodies (NBs; [68, 69]; Fig. 2B), increased levels of inhibitors of cyclin-dependent kinases p21^WAF1/CIP1^ [70] and p16^INK4a^ ([54]; Fig. 1C) and decreased incorporation of BrdU (Fig. 2C). Overall, the patterns of these senescence markers seen in bystander cells were very similar to those of the parental senescent cells.

In our previous studies we showed that senescence-associated elevation of PML mRNA depends on autocrine/paracrine signaling mediated by the activity of STAT1 and STAT3 signaling pathways [24, 63]. Though in all three forms of “parental” senescent cells significant increase of activated forms of STAT1 (pY701), STAT3 (pY705) and STAT5 (pY694) were observed together with elevated PML protein (Suppl. Fig. 1E), surprisingly, this was not matched by the activity of the individual STAT pathways in the bystander cells (Fig. 2D). Specifically, no significant increase of STAT1 activity was found in any of the three forms of bystander senescence by day 20, in contrast to parental senescence (Suppl. Fig. 1E). STAT5 phosphorylation was observed only in bystander cells exposed to drug-induced conditioned media, whereas pY705 STAT3 was observed after treatment with all three types of conditioned senescent medium (Fig. 2D). Also, the senescence-associated increase of plasminogen activator inhibitor-1 (PAI-1; [71]) mRNA levels was not universaly seen, being selectively associated only with replicative senescence in both parental and bystander senescent cells (Fig. 2E, F).

Importantly, however, the exposure of the U2OS tumor cell line to conditioned medium from drug-induced senescent U2OS cells did result into development of bystander senescence with expressed hallmarks of senescence, analogous to the scenario seen in normal BJ cells (Suppl. Fig. 2E-H).

To conclude, despite the partial differences among the three types of senescence-conditioned media, the senescence-associated secretome of cells undergoing any of the three forms of parental senescence is capable of inducing durable cell cycle arrest with hallmarks of ‘bystander cellular senescence’ in normal human cells. In addition, the example of drug-induced parental senescence that also occurs in tumor cells, demonstrates that SAS-mediated bystander senescence can also be triggered in cancer cells.

### Reactive oxygen species contribute to SAS-induced DNA damage

**Figure 3:**
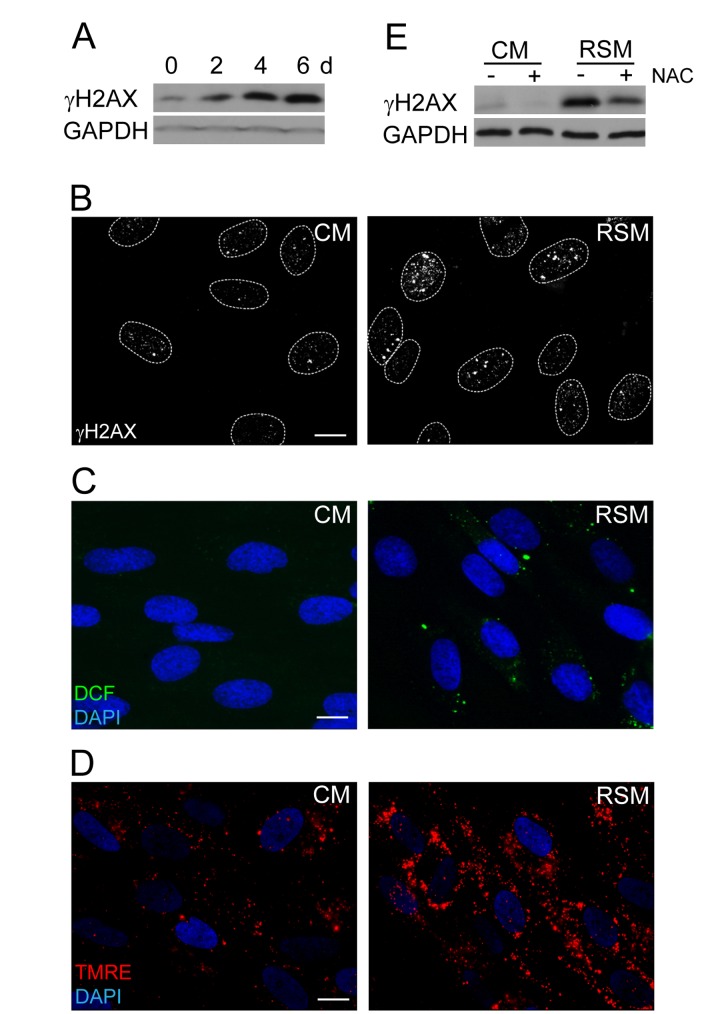
Increase of DNA damage, ROS production and mitochondrial membrane potential in cells treated with senescence-conditioned medium (**A**) Immunoblot (time course from 0 to 6 days) and (**B**) indirect immunofluorescence (day 4) detection of γH2AX in BJ cells treated with conditioned control (CM) and replicative senescent cell medium (RSM); GAPDH was used as a loading control. Bar 15 μm. (**C**) Immunofluorescence detection of ROS production by 2',7'-dichlorofluorescein (DCF) staining and (**D**) detection of mitochondrial potential by tetramethylrhodamine ethyl ester (TMRE) in BJ cells treated with conditioned control (CM) and replicative senescent cell medium (RSM). Bar 15 μm. (**E**) Immunoblot detection of γH2AX in BJ cells treated with normal or senescent medium in presence or absence of N-acetylcysteine. All these experiments except (**A**) were measured in BJ cells treated 4 days with conditioned medium from replicative senescent cells (RSM; diluted 1:1 with fresh medium). BJ cells treated 4 days with medium from non-senescent cells (CM; diluted 1:1 with fresh medium) were used as a control.

The next question we asked was whether the DNA damage observed in bystander cells (detected as increased levels of γH2AX; Fig. 3A, B) can be linked with elevated amounts of reactive oxygen species (ROS) arising as a consequence of SAS-induced changes in mitochondrial function [72]. Indeed, probing of control and bRS cells (i.e. BJ cells treated with medium from replicative senescent cells) with 2',7'-dichlorofluorescein indicated elevated levels of ROS in bRS cells (Fig. 3C). The observed enhanced ROS and DNA damage could be a consequence of increased mitochondrial potential, a scenario consistent with our measurements with TMRE (Fig. 3D). Indeed, addition of N-acetylcysteine, a scavenger of reactive oxygen radicals [73], to senescence-conditioned media significantly diminished the level of the induced γH2AX (Fig. 3E), indicating a causal link between ROS production and DNA damage observed in the bystander cells.

### IL6/STAT3 signaling is not involved in DNA damage in bystander senescent cells

**Figure 4:**
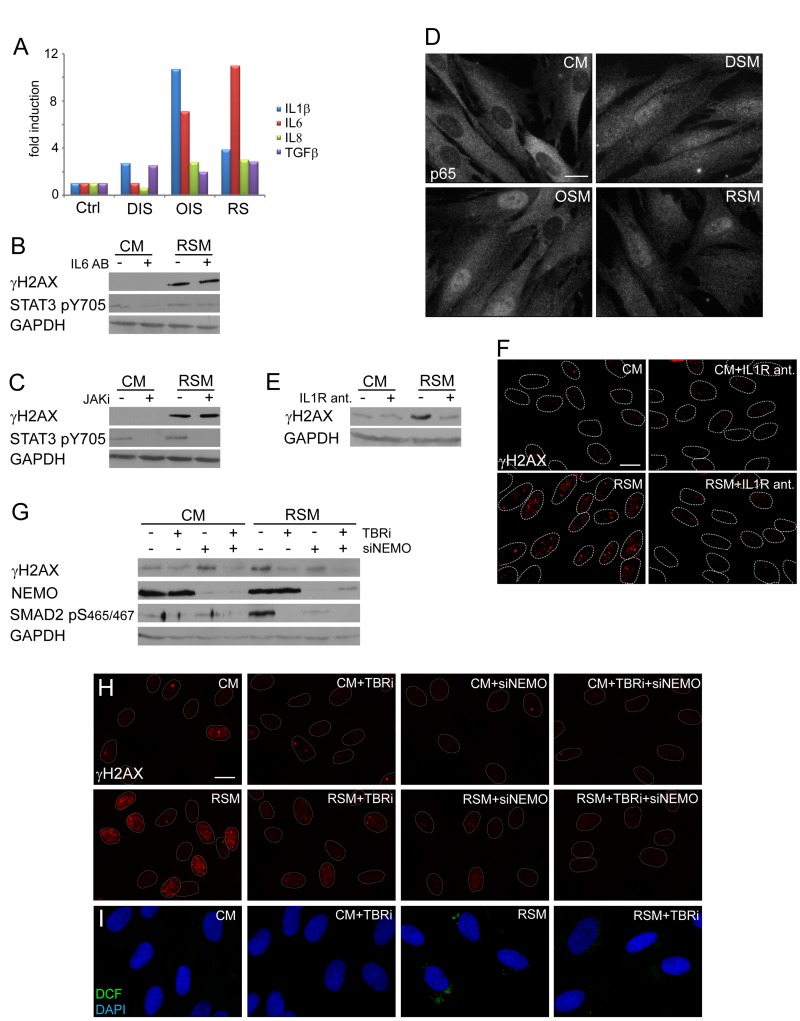
NFκB and TGFβ induce DNA damage in bystander cells (**A**) Detection of cytokines in medium of parental senescent cells using FACS beads assay. The values are shown as a fold induction relative to non-treated BJ cells (ctrl). (**B**) Immunoblot detection of serine 139 phosphorylated H2AX (γH2AX) and STAT3 phosphorylated on tyrosine 705 (STAT3 pS705) in BJ cells treated with control (CM) or replicative senescent medium (RSM) in presence or absence of IL6 neutralizing antibody (2 μg/ml). (**C**) Immunoblot detection of γH2AX and STAT3 pS705 in BJ cells treated with CM or RSM in presence or absence of JAK kinase family specific inhibitor (JAKi; 0.25 μM). (**D**) Immunofluorescence detection of the p65 subunit of NFκB in BJ cells treated with senescent medium from drug-induced (DSM), oncogene-induced (OSM) or replicative (RSM) senescent BJ cells for 4 days. BJ cells treated with medium from non-senescent BJ cells (CM) were used as a control. Bar 15μm. (**E**) Immunoblot and (**F**) indirect immunofluorescence detection of γH2AX in BJ cells treated with CM or RSM in presence or absence of IL1 receptor antagonist (IL1R ant.; 25 μM). Bar 15μm. (**G**) Immunoblot detection of γH2AX, NEMO and SMAD2 phosphorylated on serine 465/467 (SMAD2 pS465/467) and (**H**) immunofluorescence detection of γH2AX foci in BJ cells treated with CM or RSM in presence or absence of TGFβ receptor inhibitor (TBRi; 10 μM), NEMO siRNA or combination of both. Cells without transfection with siNEMO were transfected with control siRNA. Bar 15μm. (**I**) Detection of ROS production by 2',7'-dichlorofluorescein (DCF) staining in BJ cells treated with CM or RSM in presence or absence of TGFβ receptor inhibitor (TBRi; 10 μM). Bar 15μm. All these experiments (except experiment **A** and **C**) were measured in BJ cells treated 4 days with conditioned medium from replicative senescent cells (RSM; diluted 1:1 with fresh medium). BJ cells treated 4 days with medium from non-senescent cells (CM; diluted 1:1 with fresh medium) were used as a control.

Next we assessed which component(s) of the senescence-associated secretome is involved in DNA damaging activity of senescence-conditioned media. Kuilman et al. reported direct involvement of autocrine IL6/STAT3 signaling in promotion and maintenance of ‘primary’ OIS [20]. As culture media conditioned by all three forms of senescence contained elevated levels of IL6 (Fig. 4A), we tried to inhibit the activity of the IL6/STAT3 signaling pathway in bystander cells by IL6 neutralizing antibodies or through inhibition of STAT3 activating kinases JAK by a specific chemical inhibitor (iJAK) and monitored the resulting amounts of the nuclear γH2AX foci induced in bystander cells. However, no significant effect on the number of γH2AX foci was observed in bRS BJ cells irrespective of the used approach of STAT3 signaling inhibition (see Fig. 4B, C). The ability of IL6 neutralizing antibodies to inhibit IL6 biological (growth-promoting) activity was verified, using methods published in our previous studies ([24, 74]; see Suppl. Fig. 1F). These results indicate that the IL6/STAT3 signaling pathway does not directly contribute to the observed DNA damaging activity of senescence-conditioned media.

### IL1 and TGFβ induce Nox4 and promote DNA damage in bystander senescent cells

Proinflammatory cytokines including IL1β can trigger production of ROS (see e.g. [75]). Both parental and bystander senescent BJ cells irrespective of senescence the initial promoting mechanism express and secrete IL1β (see Table 1 and Fig. 4A). Since IL1β was described as a strong activator of NFκB signaling [76], we compared the subcellular distribution of the p65 subunit of NFκB in replicative, oncogene- and drug-induced bystander senescent cells relative to control non-senescent cells. As shown on Fig. 4D (see also Suppl. Fig. 2I for U2OS), all three forms of senescent cells show redistribution of p65 from cytosol into the nucleus indicative of activation of the NFκB signaling pathway in bystander cells. Inhibition of IL1 receptor signaling using IL1 receptor antagonist led to a significant reduction of γH2AX levels (Fig. 4E) and γH2AX foci (Fig. 4F) in bRS BJ cells. Moreover, siRNA-mediated knockdown of NEMO/IKKγ subunits of the NFκB-activating signalosome complex necessary for NFκB activation [77] resulted in partial decrease of γH2AX levels (Fig. 4G) and γH2AX foci in bRS BJ cells (Fig. 4H) supporting the involvement of IL1/NFκB pathway in DNA DSB formation in bystander senescent cells.

All three forms of parental senescent cells secreted high levels of TGFb1 (see Fig. 4A), the cytokine known to induce or reinforce senescence [78-80], and as such another candidate to trigger DDR in bystander cells. The inhibition of TGFβ signaling, that was otherwise strongly activated in bRS cells (detected as phosphorylation of SMAD2 at serine 465/467; [81]; see Fig. 4G), with a TGFβ receptor inhibitor resulted in reduction of γH2AX levels (Fig. 4G) and decreased numbers and intensity of γH2AX foci (Fig. 4H), as well as in reduction of ROS production (Fig. 4I). Furthermore, the combined inhibition of both TGFβ and NFκB signaling completely suppressed γH2AX levels and DNA damage foci formation in bRS cells to levels observed in control, proliferating cells (Fig. 4G, H).

**Table 1:** Detection of cytokine mRNA expression in parental and bystander senescent cells

Cytokine	DIS-BJ		OIS-BJ		RS-BJ		DIS-U2OS	
	Parental	SASP-induced	Parental	SASP-induced	Parental	SASP-induced	Parental	SASP-induced
**IFNG**	−1.05	**-4.41**	**-3.05**	−1.27	−1.35	1.53	**4.57**	−1.72
**IL1A**	**2.66**	**12.68**	**10.91**	**3.69**	**4.65**	**5.39**	**33.26**	1.33
**IL1B**	**1.85**	**6.54**	**25.71**	**6.19**	**2.64**	**5.86**	**75.74**	−1.57
**IL6**	−1.16	**2.42**	**6.98**	**3.40**	**3.57**	**3.10**	**80.70**	**19.13**
**IL8**	**-3.64**	**-2.64**	**21.91**	**3.67**	**5.43**	**1.88**	**532.94**	**81.46**
**TNF**	−1.27	**-2.22**	**-3.74**	−1.43	−1.94	**1.87**	**886.35**	**18.10**

These results indicate that TGFb and NFκB signaling pathways together induce DNA damage foci formation in bystander senescent cells.

**Figure 5:**
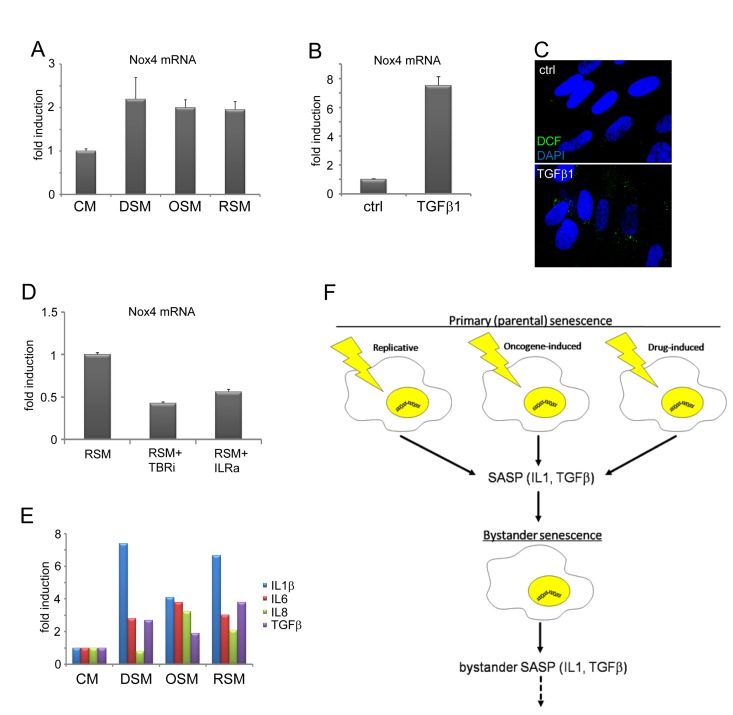
TGFβ- and IL1-dependent expression of Nox4 in bystander senescent cells (**A**) Nox4 mRNA levels quantified by real time qRT-PCR in BJ cells treated with senescent medium from drug-induced (DSM), oncogene-induced (OSM) or replicative (RSM) senescent BJ cells for 20 days or (**B**) treated with recombinant TGFβ1 (1μM) for 4 days. BJ cells treated with medium from non-senescent BJ cells (CM) or non-treated cells (ctrl) were used as a control. The mRNA values represent average of two independent experiments and are shown as a fold induction relative to control BJ cells; error bars represent standard error. β-actin was used as a reference gene. (**C**) Detection of ROS production by 2',7'-dichlorofluorescein (DCF) staining in BJ cells in presence or absence of recombinant TGFβ, protein (4 days). (**D**) Nox4 mRNA levels quantified by real time qRT-PCR in BJ cells treated with replicative senescent cell medium (RSM) in presence or absence of TGFβ receptor inhibitor (TBRi; 10 μM) or IL1 receptor antagonist (IL1R ant.; 25 μM). The mRNA values represent average of two independent experiments and are shown as a fold induction relative to RSM BJ cells; error bars represent standard error. β-actin was used as a reference gene. (**E**) Detection of cytokines in medium of different bystander senescent cells 20 days after treatment using FACS beads assay. Fresh medium was added 24 hours before harvest to allow measurement of cytokines produced by bystander cells. The values are shown as a fold induction relative to BJ cells treated with medium from non-senescent BJ cells (CM). (**F**) Schematic representation of the IL1- and TGFβ-dependent induction of DNA damage and secondary (bystander) senescence common to three forms of primary (parental) senescence: senescence-associated secretome (SASP), especially IL1β and TGFβ, produced by three forms of senescent cells is able to induce DNA damage and bystander senescence in neighboring cells. Induction of secondary SASP in bystander cells indicates a possibility to spread DNA damage and senescence in surrounding tissue.

Weyemi at al. found that NADPH oxidase Nox4 is responsible for DNA damage during H-Ras^V12^-induced senescence [82]. Besides mitochondria, membrane localized NADPH oxidases including Nox4 serve as an alternative source of intracellular ROS production [83-85]. Notably, both IL1 [86] and TGFb [87, 88] can induce Nox4 expression. Indeed, the expression of Nox4 mRNA was elevated in all three forms of bystander senescence (Fig. 5A) and it was TGFb-inducible in control BJ cells (Fig. 5B). The treatment of control BJ cells with TGFb also resulted into increased ROS production (Fig. 5C). Moreover, inhibition of either TGFb or IL1 receptor suppressed the level of Nox4 mRNA in cells exposed to medium conditioned by replicative senescent cells (Fig. 5D).

Taken together, the DNA damage in bystander cells was induced by additive effects of TGFβ and IL1 signaling pathways and the expression of NADPH oxidase Nox4 is a candidate mediator to trigger TGFβ- and IL1-dependent DNA damage in bystander cells.

### Induction of senescence-associated cytokine expression in bystander cells

Provided the SAS-induced senescence might occur also in vivo, the important question emerges whether the secondary senescent bystander cells can further promote the premature senescence far from primary focus by producing their own SAS. Therefore we asked, whether bystander senescent cells also possess SAS and, if so, what is its character/composition in relation to parental senescent SAS, and whether it is dependent on the primary senescence-inducing stimulus. For this purpose we compared cytokine expression in DIS, OIS and RS and their respective SAS-induced senescent bystanders. We estimated the levels of six selected cytokines known to be associated with primary parental senescence, and either capable of inducing a production of DNA damaging ROS (IL1a, IL1β, IFNγ and TNFα; see [75]) or being ROS-inducible (IL6 and IL8; see [89, 90]), in culture media conditioned by three forms of the parental senescent cells (see Material and Methods for details). To compare the potential production of the same set of cytokines by ‘bystander’ senescent cells, conditioned culture medium was removed at day 20 and substituted with fresh culture medium. Cells were then cultivated for another 24 hours and mRNA levels in cell lysates (Table 1) or concentration of cytokine polypeptides released into the medium were estimated (Fig. 5E). IL1a (mRNA) andIL1β (both mRNA and protein) were increased in all three forms of parental as well as bystander senescence in normal diploid BJ fibroblasts, but not in drug-induced U2OS (sarcoma) bystander senescent cells. IL6 and IL8 were not increased in drug-induced parental or bystander BJ cells but were elevated in oncogene-induced and replicative parental and bystander senescent BJ cell and drug-induced senescent U2OS (again both parental and bystander). There was no induction of IFNγ expression in any type of parental or bystander normal BJ cells, but there was an increase in parental drug-induced senescent U2OS tumor cells, which correlates with increase of IFNγ secretion in this cell line. TNFα was elevated only in parental and bystander DIS U2OS cells. Notably, TGFβ was secreted by all forms of bystander senescent cells (Fig. 5E).

Collectively, our data show that activation of cytokine expression characteristic for cellular senescence is a component of bystander senescent cell phenotype as well, and may be spread from cell to cell. Importantly, the ROS-inducing cytokines IL1β and TGFβ were produced also by bystander cells, suggesting a potential for spreading of their biological effects to cells more distant from those directly exposed to the initial senescence-inducing insult.

## DISCUSSION

The enhanced secretion of various substances including cytokines is a characteristic feature shared by various forms of cellular senescence-inducing autocrine and paracrine effects in the vicinity of senescent cells [48, 49]. On the other hand, it remains relatively poorly defined whether and how the nature of the senescent secretome and thus its (patho)physiological effects depend on the cell type and the nature of the senescence inducing stimulus. Although some cytokine species are only variably present in SAS (our unpublished data and Fig. 5E), it seems that some proinflammatory cytokines are commonly present in various forms of senescence. These shared, non-variant species are therefore candidate universal effectors of the senescence-associated secretome that can induce bystander senescence in a paracrine manner. In this study we showed that cells undergoing primary (parental) replicative, oncogene- and drug-induced senescence secrete factors competent to induce enhanced ROS production, DNA damage response and, indeed, paracrine cellular senescence in normal human fibroblasts. By manipulating the signaling pathways of IL6/STAT3, IL1β/NFκB and TGFβ/SMAD, i.e. cascades that are commonly activated in these three forms of senescence, we found that the latter two are required for, and cooperate to enhance ROS production and fuel the DNA damage response observed in bystander senescent cells.

### The DNA damage and senescence-inducing activity of SAS

Notably, the culture media conditioned by any of the three types of primary/parental senescent cells were capable of activating the ATM/Chk2/p53 axis of the DNA DSB response in normal cells. This is in agreement with current view that cellular senescence is triggered and maintained by persistent DNA damage signaling [65, 91] and with the work published by Nelson et al. showing the activation of the DDR and presence of DNA damage foci in MRC5 fibroblasts induced to senescence by conditioned medium of replicatively senescent MRC5 cells [49]. As we observed, the onset of DDR activity in bystander cells was relatively fast, detectable already after 48 hours of exposure to senescence-conditioned medium, suggesting direct involvement of DNA damage check-point(s) in development of such paracrine bystander senescence. Although we did not fully elucidate the precise cause and nature of the DNA damage in bystander cells, our data implicate DNA DSB formation (indicated by ATM/Chk2 activation and γH2AX foci formation), and the observed decrease of DDR markers upon reactive oxygen radical scavenger N-acetylcysteine indicated the participation of ROS. These results indicates that ROS participate both in primary senescence, as documented for oncogene-induced senescence [92, 93], and secondary-bystander senescence. Importantly, data obtained by us (this study) and others [49] underscore the role of secreted cytokines both in bystander senescence but also in primary senescence. As the secretome of senescent cells is rich in diverse cytokine species, it is challenging to identify the key cytokine species causally linked to the senescence phenotype. Based on the previous studies [20, 21, 24] we proposed a model of senescence initiated and maintained by cytokine-driven signaling loops operating in mutually linked positive feedbacks [94] that further complicate the identification of those cytokine(s) involved in the initial phases of senescence.

Kojima et al. recently described the ability of the IL6 pathway to induce ROS production and senescence in fibroblasts via activation of insulin-like growth factor-binding protein 5 (IGFBP5) [45]. Furthermore, the IL6/STAT3 pathway is involved in control of mitochondrial oxidative phosphorylation and mito-chondrial membrane potential [95], which might explain the observed increase of ROS production and changes in mitochondrial membrane potential in bystander cells by IL6 produced by primary senescent cells. Though we observed the increase of serine 727-phosphorylated form of STAT3 in bystander cells that has been reported to enter mitochondria and modulate the activity of electron transport chain complexes I and II [95-97], we were unable to detect any significantly higher levels of STAT3 in mitochondria of senescent cells (data not shown). Moreover, neutralization of IL6 with specific antibodies or chemical inhibition of JAK kinases in our present experiments failed to exert any effect on the level of ROS (not shown) and extent of DDR in bystander senescent BJ fibroblasts, therefore not supporting the role of IL6/STAT3 signaling in enhanced ROS production and elevation of DDR in bystander BJ cells.

Our analysis of cytokines produced by parental and bystander senescent BJ cells revealed further candidate species with known genotoxic activity. IL1α and IL1β were invariably secreted at higher levels in both parental and even bystander senescent BJ cells. Both IL1α and IL1β have been reported to play a pivotal role in induction of other cytokines associated with senescence, such as IL6 and IL8, effects mediated by activity of NFκB (see, e.g. [90, 98], for a review of the role of NFκB in senescence, see, e.g. [99]). Our data indicated that IL1b-inducedROS production [90] contributed to the onset of DDR in bystander cells, since inhibition of IL1 receptor or suppression of NFκB activation by knockdown of NEMO/IKKγ decreased significantly, though not completely, the level of DDR markers in bystander cells. The mechanism of IL1-dependent induction of ROS and DDR in bystander cells is not known. Previous studies on biological effects of IL1 showed that IL1 is able to induce expression of Nox4 gene in human coronary artery smooth muscle cells [86]. Nox4 is a member of NADPH oxidase NOX/DUOX family known to regulate production of ROS, especially superoxide forms [83], to induce DNA damage, genomic instability [100, 101] and premature cellular senescence in endothelial cells [102, 103]. Importantly, Weyemi et al. described a role of Nox4 in H-RasV12-induced replication stress, cell cycle arrest and development of senescence in human thyroid cells, as knockdown of Nox4 resulted in suppression of ROS production, expression of cdc6 (a regulator of DNA replication), DNA damage and development of senescence [82]. It is possible that the effect of activated oncogene on Nox4 expression reported in the study of Weyemi et al. is at least in part mediated secondarily by autocrine/paracrine effects of secreted cytokines. Lu et al. described direct binding of NFκB on the Nox4 promoter and activation of its expression [84], underscoring the role of NFκB activating cytokines in Nox4 induction, increase of superoxide radicals and induction of DNA damage. Thus, NFκB activation triggered by upstream cytokine signaling pathways may represent an important upstream trigger of the complex cascade of events promoting senescence.

The enhanced expression of members of the TGFb superfamily are frequently found in expression profiles of senescent cells [18, 24, 104, 105]. Activation of TGFβ signaling results in SMAD2 and SMAD3 phosphorylation and their hetero-trimerization with the SMAD4 coactivator. Relocalization of the SMAD2/3/4 complex from cytoplasm into nucleus triggers expression of many genes including those linked to cell cycle arrest (for a review, see [106]). It was found that TGFβ1-dependent growth arrest in G1 phase is accompanied by increased levels of p15^INK4B^, p16^INK4A^ and activation of p53 [107] and depletion of TGFβ from culture medium results in constitutive induction of CDK2 and CDK4 kinase activity and Rb phosphorylation in mouse keratinocytes [108]. Importantly, ectopic expression or administration of TGFb is capable of inducing premature senescence in several cell types, such as human mammary epithelial stem cells [78, 109], human lung adenocarcinoma cells [79, 110], hepatocellular carcinoma cells [111] and prostate epithelial cells [112]. Abrogated TGFβ signaling can bypass replicative [113], oncogene-induced [108, 114], and H_2_O_2_-induced senescence [115]. Interestingly, cytoplasmic PML isoform seems to mediate the TGFb-dependent cell cycle arrest accompanying senescence [116]. Yoon et al. reported that TGFβ1 arrested lung epithelial cells at G1 phase by prolonged generation of ROS accompanied with decreased activity of complex IV of mitochondrial respiratory chain [117]. Notably like IL1, TGFβ was found to elevate expression of Nox4 gene [87, 88]. Although experimental proof for a direct link between TGFβ and NFκB-mediated Nox4 expression remains to be provided, the ability of TGFβto activate NFκB [118] suggests this possibility. All these data support the role of TGFβ signaling in development of DDR and bystander senescence observed by us. As we found, the medium conditioned by cells undergoing any of the three forms of primary senescence contains elevated levels of TGFβ. Moreover, the activation of TGFβ pathway detected as phosphorylated SMAD2 was observed in bystander cells. Inhibition of TGFβ receptor by specific inhibitor led to partial decrease of ROS production as well as the extent of DDR. Thus TGFb production by primary senescent cells can causally contribute to cell cycle arrest associated with secondary bystander senescence. Importantly, simulta-neous inhibition of TGFb signaling and NFκB led to suppression of DDR to the levels in control cells indicating that these two pathways play additive roles in fueling the activation of DDR in bystander senescent cells.

To conclude, secretome associated with three major forms of cellular senescence is able to activate the DNA damage response pathway and senescence-associated cell cycle arrest in neighboring cells in vitro in a paracrine manner (see scheme Fig. 5F). At the conceptual level, we propose that the observed induction of ROS, through its emerging proliferation-promoting effects (see [82], for discussion) could also contribute to the replication stress known to underlie the oncogene-induced senescence [5, 6]. In other words, we propose the presence, and biological impact, of the secreted IL1 and TGFb, along with Nox4 signaling, as the candidate unifying mechanism that triggers the DDR signaling in all major forms of bystander senescence. Before further evaluation of the potential pathophysiological role of this concept, it will be necessary to prove that similar TGFβ-and IL1-mediated genotoxic effects take place also in vivo at sites of senescent cell accumulations. Provided this concept is validated under in vivo conditions, our present results would help to explain for example the contribution of senescent cells to age-associated inflammation (inflammaging, see [119]) responsible for age-related inflammatory degenerative diseases, such as atherosclerosis, where the role of inflammatory cytokines [120] and TGFβ[121] has been already reported. Another intriguing question originating from our study is whether the secondary (and tertiary) SASP (Fig. 5F) possess DNA damaging and senescence-inducing activity, which can be responsible for spreading of DNA damaging activity in tissues surrounding senescent cells.

## MATERIALS AND METHODS

### Chemicals and antibodies

JAK inhibitor I, TGF beta receptor 1 inhibitor II and IL1 receptor antagonist were purchased from Merck KGaA (Darmstadt, Germany). The following antibodies were used for immunoblot: rabbit polyclonal antibodies against PML, STAT3 (clone C-20), NEMO, total Chk2, p53 and p16, mouse monoclonal antibody against p21 all from Santa Cruz Biotechnology (Santa Cruz, CA, USA), mouse monoclonal antibody against phosphotyrosine 705 of STAT3, rabbit polyclonal antibodies against phosphotyrosine 701 of STAT1, phosphoserine 727 of STAT3, phosphoserine 15 of p53, phosphothreonine 68 of Chk2, phosphoserine 465/467 of SMAD2, total STAT5 and phosphotyrosine 694 of STAT5 all from Cell Signaling Technology (Danvers, MA, USA), mouse monoclonal antibody against GAPDH (GeneTEX, Irvine, CA, USA), mouse monoclonal antibody against phosphoserine 139 of histon H2AX (Millipore, Billerica, MA, USA), mouse monoclonal antibody against Rb (BD Biosciences), mouse monoclonal antibody against H-RAS (Calbiochem) and mouse monoclonal antibody against total STAT1 (SM2 clone, Exbio, Vestec, Czech Republic).

The following antibodies were used for indirect fluorescence: mouse monoclonal antibody PG-M3 against PML, rabbit polyclonal antibodies against p65 and 53BP1 all from Santa Cruz Biotechnology (Santa Cruz, CA, USA), mouse monoclonal antibody against phosphoserine 139 of histone H2AX (Millipore, Billerica, MA, USA), rabbit polyclonal antibody against phosphoserine 139 of histone H2AX, mouse monoclonal antibody against phosphoserine 1981 of ATM, all from Cell Signaling Technology (Danvers, MA, USA). For immunofluorescence, secondary antibodies anti-mouse IgG antibody conjugated with Cy3 (Jackson ImmunoResearch Laboratories, West Grove, PA, USA) and anti-rabbit IgG antibody Alexa 488 (Invitrogen, Carlsbad, CA, USA) were used.

### Cell cultures

Human cancer cell lines U2OS (osteosarcoma) and normal human fibroblasts BJ at population doublings 30 - 35 (young) and 80 (senescent) were cultured in Dulbecco's modified Eagle's medium (D-MEM) supplemented with 10% foetal bovine serum (FBS). Cells were kept at 37°C under 5% CO_2_ atmosphere and 95% humidity.

### Induction of bystander senescence

Throughout this study, medium conditioned by young (control) or “parental” senescent cells were used to induce “bystander” senescence. Drug-induced senescence (DIS) was induced by 10 μM etoposide applied for 48 hours, the medium was then replaced with fresh medium and cells were cultivated for other 6 days to achieve senescence. At day 8, fresh medium was added and cells were cultivated for 24 hours to condition the medium with cytokines. Collected drug-induced conditioned medium (DSM) was centrifuged (2000 rpm/5 min), filtered through 0.2 μm filter, diluted 1:1 with fresh medium and used for cultivation of young BJ cells. Replicative senescent BJ fibroblasts at population doubling 80 were used to condition replicative senescence medium (RSM). Again, replicative senescent cells were cultivated for 24 hours in fresh medium to prepare RSM as was described above. Oncogene-induced senescent BJ cells stably transfected with tetracycline-induced constitutively active form of RAS (H-Ras^V12E^; [54]) were used for preparation of oncogene-induced senescent medium (OSM; [24, 122]). Cells were incubated with doxycyclin for 16 days to activate RAS expression and senescence. At this time, conditioned medium was prepared as was described above. Control medium (CM) for replicative and drug-induced senescence was collected from normal BJ cells after 24 hours from the fresh medium was added. Control medium for oncogene-induced senescence was obtained from BJ cells transfected with empty vector. For long term experiments, control and senescent media were aliquoted and frozen in -80°C until use.

### Indirect immunofluorescence

Cells grown on glass coverslips were fixed by 4% formaldehyde and permeabilized by 0.1% Triton X-100 in two consecutive steps, each for 15 minutes at RT. After washing with PBS, cells were incubated in 10% FBS (diluted in PBS) for 30 min to block unspecific signal. After this step cells were incubated with diluted primary antibodies for 1 hour at RT and then extensively washed with PBS/0.1% Tween 20. The incubation with secondary antibodies was performed for 1 hour at RT. To counterstain nuclei, coverslips were mounted in Mowiol containing 4',6-diamidino- 2-phenylindole (DAPI; Sigma, St. Louis, MO, USA) and viewed by a fluorescence microscope (Leica DMRXA, Germany). For detection of PML and 53BP1 colocalization, confocal microscope was used (Leica TCS SP, Weltzlar, Germany).

### Quantification of DNA damage foci and BrdU positive cells

53BP1 DNA damage foci were counted on images obtained using a fluorescence microscope (Leica DMRXA, Germany); 400-500 cell nuclei were counted per sample. Quantification of BrdU-positive cells was done as described [122]; 700 - 1000 cells were counted per sample.

### Detection of ROS and mitochondrial potential by fluorescent probes

Cells grown on glass coverslips were incubated for 15 minutes with 50 μM 2?,7?-dichlorofluorescein (Sigma, St. Louis, MO, USA) for ROS detection or with 1.5 μM tetramethylrhodamine ethyl ester (TMRE, Invitrogen, Carlsbad, CA, USA)to detect mitochondrial potential. After fixation with 4% formaldehyde, coverslips were mounted in Mowiol containing DAPI to counterstain nuclei and viewed by the fluorescence microscope (Leica DMRXA, Germany).

### Quantitative real time RT-PCR (qRT-PCR)

Total RNA samples were isolated using RNeasy Mini Kit (Qiagen, MD, USA) as according to the manufacturer's protocol. First strand cDNA was synthesized from 200 ng of total RNA with random hexamer primers using TaqMan Reverse Transcription Reagents (Applied Biosystems). qRT-PCR was performed in ABI Prism 7300 (Applied Biosystems) using SYBR Green I Master Mix (Applied Biosystems). For detection of cytokine expression, human common cytokines PCR array (SA Bioscience, Valencia, CA, USA) was used. Data from PCR array was verified with the following set of primers: **IL6**: 5'-AGA CAG CCA CTC ACC TCT TCA G -3', 5'-TTC TGC CAG TGC CTC TTT GCT G-3'; **IL8**: 5'-TTG GCA GCC TTC CTG ATT TC-3', 5'-TCT TTA GCA CTC CTT GGC AAA AC-3'; **IL1 α**: 5'-TGT ATG TGA CTG CCC AAG ATG AAG-3', 5'-AGA GGA GGT TGG TCT CAC TAC C-3'; **IL1 β**: 5'-CCA CAG ACC TTC CAG GAG AAT G-3', 5'-GTG CAG TTC AGT GAT CGT ACA GG-3'; **TNF α**: 5'-CTC TTC TGC CTG CTG CAC TTT G-3', 5'-ATG GGC TAC AGG CTT GTC ACT C-3'; **PAI**: 5'-CTC ATC AGC CAC TGG AAA GGC A-3', 5'-GAC TCG TGA AGT CAG CCT GAA AC-3'; **Nox4**: 5'- GCC AGA GTA TCA CTA CCT CCA C-3', 5'- CTC GGA GGT AAG CCA AGA GTG T-3'; **β-actin**: 5'-CCA ACC GCG AGA AGA TGA-3', 5'-CCA GAG GCG TAC AGG GAT AG-3'. The relative quantity of cDNA was estimated by ΔΔCt [123], data were normalized to β-actin. Samples were measured in triplicates.

### SDS-PAGE and immunoblotting

Cells were harvested into Laemmli SDS sample lysis buffer, sonicated and centrifuged at 13,200 rpm for 10 min. Concentration of proteins was estimated by the BCA method (Pierce Biotechnology Inc., Rockford, USA). 100 mM DTT and 0.01% bromphenol was added to lysates before separation by SDS-PAGE (9 and 14% gels were used). The same protein amount (25 μg for BJ cells, 35 μg for U2OS cell line) was loaded into each well. Proteins were electrotransferred onto a nitrocellulose membrane using wet transfer and detected by specific antibodies combined with horseradish peroxidase-conjugated secondary antibodies (goat anti-rabbit, goat anti-mouse, Bio-Rad, Hercules, CA, USA). Peroxidase activity was detected by ECL (Pierce Biotechnology Inc.). GAPDH was used as a marker of equal loading.

### Determination of cytokines in cultivation media

The conditioned medium from cells was collected 24 hours after fresh medium was changed and the numbers of cells per each dish were counted. The concentration of cytokines were estimated by ‘FACS bead array’ using FlowCytomix Human Simplex Kit (IL6, BMS8213FF; IL1β, BMS8224FF; IL8, BMS8204FF; TNFα, BMS8223FF; IFNg, BMS8228FF; TGFβ1, BMS8249FF; Bender MedSystems, Wien, Austria) on flow cytometer LSRII (BD Biosciences, San Jose, USA) according to manufacturer's protocol.

### Estimation of IL6 biological activity

To test effectiveness of IL6 depletion mediated via IL6 antibody (2 μg/ml; goat polyclonal antibody, R&D Systems, Inc., Minneapolis, Minnesota, USA), growth dependency of mouse hybridoma B9 cells on presence of IL6 was utilized [124]. The conditioned media from BJ cells incubated for 4 days with IL6 antibody were transferred in 1:1 dilution with fresh medium to mouse hybridoma B9 cells seeded in triplicate at density 25 000 cells/ml on 24 well plate. As positive or negative controls, B9 cells were cultivated with or without addition of recombinant IL6 (100 pg/ml), respectively. 50 μl aliquots of B9 cell cultures were removed after 3 days and cell growth and viability were measured after staining with Hoechst 33258 (Invitrogen, Carlsbad, CA, USA) by flow cytometer (BD LSRII, BD, Franklin Lakes, NJ, USA).

### siRNA-mediated gene knock-down

Specific siRNAs were introduced into cells using Lipofectamine™ RNAiMAX (Invitrogen, Carlsbad, CA, USA). Nonsense siRNA sequences (siNC; Ambion, CA, USA) were used as a negative control siRNA. siRNA against NEMO/IKKγ (Dharmacon, CO, USA) was mix of four siRNA, no sequence was available.

## SUPPLEMENTARY FIGURES



## Abbreviations

bDIS: “bystander” drug-induced senescence
bOIS: “bystander” oncogene-induced senescence
bRS: “bystander” replicative senescence
DAPI: 4’,6-diamidino-2-phenylindole
DDR: DNA damage response
GAPDH: glyceraldehyde 3-phosphate dehydrogenase
DCF: 2’,7’-dichlorofluorescein
IFN: interferon
IKK: IκB kinase
IL: interleukin
JAK: Janus kinase/just another kinase
NFκB: nuclear factor kappa B
pDIS: “parental” drug-induced senescence
pOIS: “parental” oncogene-induced senescence
pRS: “parental” replicative senescence
PML: promyelocytic leukemia protein
PML NBs: promyelocytic leukemia nuclear bodies
ROS: reactive oxygen species
SA-β-gal: senescence-associated beta-galactosidase;
SAS: senescence-associated secretome
STAT: signal transducers and activators of transcription
TGFβ: transforming growth factor beta
TNFα: tumor necrosis factor alpha
TMRE: tetramethylrhodamine ethyl ester
