# Supplementary material for: IL1- and TGFβ-Nox4 signaling, oxidative stress and DNA damage response are shared features of replicative, oncogene-induced, and drug-induced paracrine ‘Bystander senescence’
Source: Aging (Albany NY). 2012 Dec 30;4(12):932–51. doi: 10.18632/aging.100520 (PMC3615160; doi:10.18632/aging.100520)
Supplement: Supplementary file 1 [file aging-04-932-s001.pdf]

SUPPLEMENTARY FIGURES

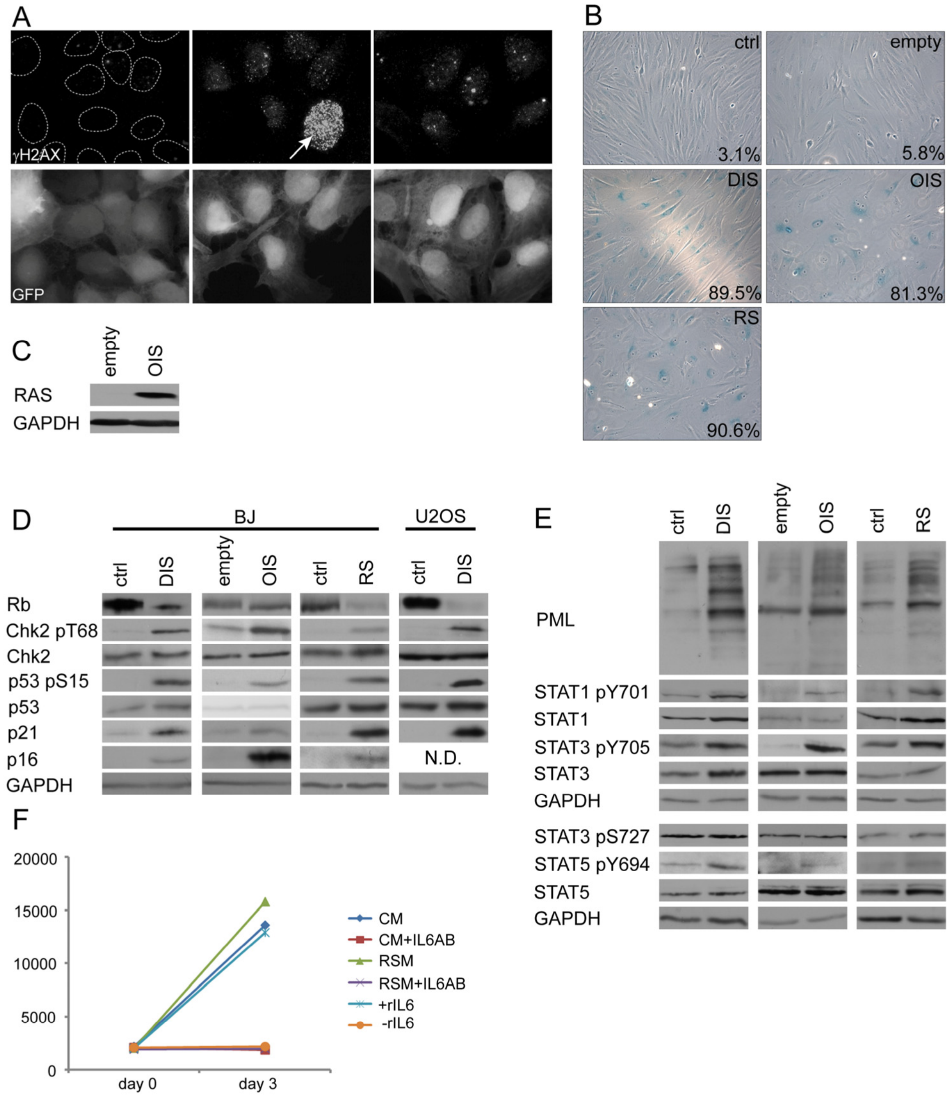

Supplementary Figure 1. (**A**) Immunofluorescence detection of γH2AX foci in non-treated GFP-U2OS cells (left panel), untreated GFP-U2OS cells mixed with drug-induced U2OS senescent cells in close contact (middle panel; senescent cell is marked with arrow), and distant (right panel). Bar 15μm (**B**) Senescence-associated β-galactosidase detection in "parental" drug-induced (DIS), oncogene-induced (OIS) and replicative senescent cells (RS) in comparison to control BJ cells (ctrl) or cells transfected with empty vector (empty). (**C**) Immunoblot detection of H-RAS expression in oncogene-induced senescent BJ cells in comparison to BJ cells transfected with empty vector. GAPDH was used as the loading control. (**D**) Immunoblot detection of Rb, p21, p16, total p53 and Chk2, serine 15 phosphorylated p53 (p53 pS15) and threonine 68 phosphorylated Chk2 (Chk2 pT68) in different types of senescent BJ cells. GAPDH was used as a loading control. (**E**) Immunoblot detection of PML, total STAT1, STAT3 and STAT5, tyrosine 701 and serine 727phosphorylated STAT1, tyrosine 705 and serine 727 phosphorylated STAT3 and tyrosine 694 phosphorylated STAT5 in replicative (RS), H-RasV12-induced (OIS) and drug-induced (DIS) BJ cells. GAPDH was used as a loading control. (**F**) IL6-dependent proliferation assay of B9 mouse hybridoma cells. Estimation of IL6 activity in medium conditioned by normal BJ cells (CM) or cells treated with medium from replicative senescent cells (RSM) two days without or with IL6 depletion using IL6 antibody (2 μg/ml). B9 cells treated with recombinant human rhIL6 (100 pg/ml) were used as a positive control (+rhIL6), B9 without rhIL6 addition were used as a negative control (-rhIL6).

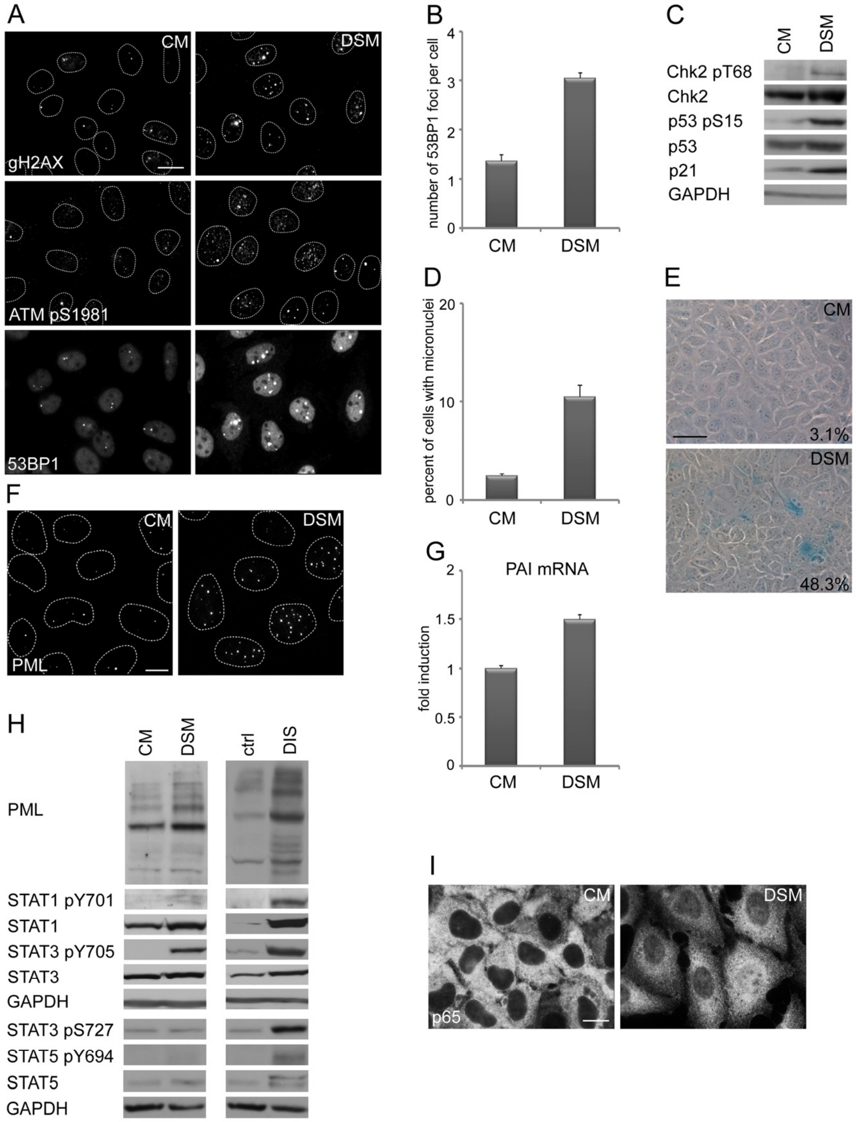

Supplementary Figure 2. Drug-induced bystander senescence in U2OS cells. Immunofluorescence detection of 53BP1, γH2AX, serine 1981 phosphorylated ATM (**A**) and quantification of numbers of 53BP1 foci (**B**) in U2OS cells treated 20 days with control (CM) or medium conditioned by drug-induced senescent cells (DSM). Bar 15 μm. (**C**) Immunoblot detection of Rb, p21, p16, total p53 and Chk2, serine 15 phosphorylated p53 and threonine 68 phosphorylated Chk2 in U2OS cells treated as in A. GAPDH was used as a loading control. (**D**) Quantification of numbers of micronuclei and (**E**) detection of senescence-associated β-galactosidase in U2OS cells treated 20 days with control (CM) or medium conditioned by drug-induced senescent cells (DSM). Bar 100 μm. Immunofluorescence detection of PML nuclear bodies (PML NBs; **F**) and plasminogen activator inhibitor mRNA levels quantified by real time qRT-PCR (PAI; **G**) in U2OS cells treated as in A. The mRNA values represent average of two independent experiments and are shown as a fold induction relative to control U2OS cells (CM); error bars represent standard error. β-actin was used as a reference gene. (**H**) Immunoblot detection of PML, total STAT1, STAT3 and STAT5, phosphorylated STAT1 on tyrosine 701 and serine 727, phosphorylated STAT3 on tyrosine 705 and serine 727 and phosphorylated STAT5 on tyrosine 694. GAPDH was used as a loading control. (**I**) Immunofluorescence detection of the p65 subunit of NFκB. Bar 15 μm.
